# “Inclusive working life in Norway”: a registry-based five-year follow-up study

**DOI:** 10.1186/1745-6673-8-19

**Published:** 2013-07-08

**Authors:** Line Foss, Hans Magne Gravseth, Petter Kristensen, Bjørgulf Claussen, Ingrid Sivesind Mehlum, Knut Skyberg

**Affiliations:** 1 National Institute of Occupational Health, Oslo, Norway; 2 Department of Community Medicine, Faculty of Medicine, Institute of Health and Society, University of Oslo, Oslo, Norway

**Keywords:** Sickness absence, Sick leave, Long-term sickness absence, Work environment, Occupational factors, Intervention

## Abstract

**Background:**

In 2001, the Norwegian authorities and major labour market partners signed an agreement regarding ‘inclusive working life’ (IW), whereby companies that participate are committed to reducing sickness absence. Our main aim was to determine the effect of the IW program and work characteristics by gender on long-term (>8 weeks) sickness absence (LSA).

**Methods:**

Self-reported data on work characteristics from the Oslo Health Study were linked to registry-based data on IW status, education and LSA. From 2001–2005, 10,995 participants (5,706 women and 5,289 men) aged 30, 40, 45 and 60 years were followed. A Cox regression was used to compute hazard ratios (HR) for LSA risk. The cohort was divided into an IW group (2,733 women and 2,058 men) and non-IW group (2,973/3,231).

**Results:**

43.2% and 41.6% of women and 22.3%/24.3% of men (IW / non-IW, respectively) experienced at least one LSA. In a multivariate model, statistically significant risk factors for LSA were low education (stronger in men), shift work/night work or rotating hours (strongest in men in the non-IW group), and heavy physical work or work involving walking and lifting (men only and stronger in the non-IW group). Among men who engaged in shift work, the LSA risk was significantly lower in the IW group.

**Conclusions:**

Our results could suggest that IW companies that employ many men in shift work have implemented relevant efforts for reducing sickness absence. However, this study could not demonstrate a significant effect of the IW program on the overall LSA risk.

## Background

Developing methods to address growing challenges from long-term sickness absence is a major public health issue. The reduction in sickness absence rates has received significant attention in Europe in recent years primarily because of the high costs of sickness absence to businesses and society [1], [2]. Sickness absence represents a burden and challenge to people who wish to work, employers, the health care sector and society as a whole [3], [4]. Work environment factors are important determinants of sickness absence [5], [6] and disability pension [7]. In Norway, reducing sickness absence and disability is an important political objective. Since October 2001, these efforts are regulated through the Inclusive Working Life (IW) program, a Norwegian national intervention program implemented by authorities and major labour market partners. Under the IW agreement, participating businesses commit to working to reduce sickness absence, reduce the number of individuals leaving the labour market on a disability pension, and include the elderly and individuals with disabilities into working life [8]. The agreement has been renewed three times, most recently until 2013. As of February 2013, the agreement covers approximately 25% of all Norwegian enterprises and 57% of all employees.

The IW program represents a new approach to preventing sickness absence, which includes a closer follow-up of the cases. The solutions are anchored in the viewpoint that the workplace is an important arena for organising efforts aimed at reducing sickness absence. The government approves financial support to IW enterprises and assists in strengthening support services for any employers in need of supervision and workplace adjustment. Rather than leaving all the responsibility for health management to the physicians and patients, a dialogue between the employer and employee is fostered, and assistance from the occupational health service is sought. An evaluation of the person’s level of functioning determines how they can contribute at work, and the workplace should accommodate the person’s level of functioning.

Long-term sickness absence and disability pension may be viewed as health-related selection out of working life. Long-term sickness and disability pension both increase with age and are more frequent among individuals with a low socioeconomic position (SEP) [9], [10]. Strong associations between indicators of ill health and sickness absence have been found, particularly for longer periods of absence, and long-term absence is associated with increased mortality risk [11]. A substantial portion of individuals with long-term sickness absences never return to work but end up with a permanent disability pension [12]. Thus, understanding the reasons individuals become sick-listed is an important issue, and there is a need for more studies investigating the causes of sickness absence. To our knowledge, no studies have examined the impact of working conditions on the risk of sickness absence, including data on IW status or other similar interventions.

The objective of this longitudinal, population-based cohort study was to examine gender-specific associations between work-related factors, SEP and long-term (>8 weeks) sickness absence (LSA) in IW and non-IW persons. Our specific aim was to reveal any differences according to IW status in the associations between work characteristics and LSA and examine whether the IW program had an effect on LSA.

## Methods

The Oslo Health Study (Norwegian abbreviation: HUBRO) was conducted from 2000–2001 as a collaboration between the Norwegian Institute of Public Health, University of Oslo and Municipality of Oslo [13]. The survey included all inhabitants in Oslo aged 30, 40, 45, 60 and 75 years in 2000, for a total of 40,888 persons. The response rate was 46%, yielding a study sample of 18,770. The HUBRO survey included several questionnaires: a main questionnaire presented to all and a series of supplementary questionnaires given to different age groups containing questions on personal, social, health and work-related topics. In this study, the information regarding working conditions and health were compiled from these self-reported data and our analyses were restricted to 10, 995 respondents in the age groups of 30, 40, 45 and 60 years.

The HUBRO responses were linked to the Historical Event Database of Statistics Norway (Norwegian abbreviation: FD-Trygd) [14], which contains national social insurance information covering the entire population through several linked official registers based on a unique, 11-digit personal identification number. Data on sickness absence and education was obtained from the FD-Trygd.

In Norway, full economic compensation is given from the first day of sickness absence to persons with a pensionable income above the sickness allowance limit, which was 5,300 Euro per year as of May 2011. We collected data on the first spell of LSA (from FD-Trygd) among the 10,995 participants (5,706 women and 5,289 men) at risk of sickness absence on January 1st, 2001. Persons not at risk were excluded from the analyses. Those not at risk were defined by one of the following non-mutually exclusive conditions: death, emigration, the receipt a disability pension before the start of follow-up, a pensionable income in 2001 below the sickness absence entitlement limit, or a sickness absence on January 1st, 2001.

The Regional Committee for Medical Research Ethics Southern Norway appeoved the study.

### Study outcome

The dichotomous study outcome was having at least one spell of a long-term (>8 weeks), continuous sickness absence during the five-year period from 2001–2005. Eight weeks was chosen as the responsible doctor until 2012 was obliged to produce an “eight weeks sickness absence sertificate” including medical information and plans for treatment and rehabilitation. Therefore, sickness absence longer than eight weeks has been considered the starting point for long-term absence in Norway.

### Independent variables

The cohort was divided into IW and non-IW groups. Serial numbers from the Norwegian Labour and Welfare Administration’s IW registry were linked to individual data from HUBRO and FD-Trygd. An “IW employee” was defined as a person employed in an IW enterprise after the agreement was enacted in 2001. Following this definition, 4,791 (2,733 women and 2,058 men) were IW employees (43.6%). The remaining 2,973 women and 3,231 men were classified as non-IW employees. Data on IW status was available from March 2003.

The SEP indicator was based on the education level classifications from Statistics Norway and categorised into five levels [15], as described in the footnotes of Tables 1, 2 and 3 and in more detail in our previous studies [5], [6].

**Table 1:** **Risk of long**-**term sickness absence** (**LSA**) **according to potential determinants**

	**Women (N=5706)**				**Men (N=5289)**			
	IW** (N=2733)		**Non- IW (N=2973)**		**IW (N=2058)**		**Non- IW (N=3231)**	
	Percent of total	LSA risk	Percent of total	LSA risk	Percent of total	LSA risk	Percent of total	LSA risk
Total	100	0.432	100	0.416	100	0.223	100	0.243
Age								
30	31.0	0.485	30.9	0.459	31.1	0.138	29.9	0.174
40	26.0	0.389	22.3	0.374	24.9	0.223	21.7	0.254
45	23.4	0.399	21.2	0.374	23.2	0.262	19.4	0.254
60	19.6	0.444	25.5	0.437	20.8	0.312	29.0	0.300
Education*								
1	5.3	0.507	8.8	0.502	6.7	0.449	8.2	0.395
2	20.3	0.459	23.3	0.457	12.9	0.342	17.1	0.326
3	16.8	0.472	20.3	0.430	19.6	0.278	25.8	0.281
4	37.2	0.441	33.0	0.391	30.9	0.171	28.8	0.176
5	17.7	0.313	11.3	0.304	27.1	0.117	16.3	0.110
Missing	2.7	0.479	3.4	0.470	2.8	0.345	3.7	0.378
Industrial classification								
Secondary industry	3.7	0.363	6.1	0.383	7.7	0.302	13.9	0.267
Tertiary industry (heavy)	9.1	0.430	17.5	0.437	16.4	0.260	20.3	0.290
Office work	24.3	0.386	23.4	0.407	33.8	0.172	26.9	0.157
Teaching sector	7.7	0.386	11.6	0.391	7.4	0.203	4.7	0.255
Health sector	38.5	0.484	21.4	0.447	16.3	0.262	7.7	0.222
Missing	16.7	0.416	20.1	0.402	18.3	0.223	26.5	0.287
Shift work, night work or rotating hours?								
No	66.7	0.392	76.9	0.392	65.9	0.192	73.2	0.215
Yes	19.3	0.530	9.2	0.511	18.2	0.261	10.1	0.374
Missing	14.0	0.483	13.9	0.488	15.8	0.307	16.7	0.289
Physical job demands								
Mainly sedentary	50.9	0.383	53.4	0.380	56.0	0.162	53.7	0.176
Involving significant walking	18.9	0.462	22.7	0.432	16.7	0.271	16.3	0.294
Heavy physical work or work involving significant walking and lifting	15.8	0.506	9.1	0.507	11.1	0.364	12.8	0.403
Missing	14.3	0.483	14.8	0.469	16.3	0.287	17.1	0. 288

*Level of education was collapsed into five categories: Primary education/Lower secondary (1) Upper secondary education, basic (2) Upper secondary, final year/post-secondary non-tertiary education (3) First stage of tertiary education, undergraduate level (4) First stage of tertiary education, graduate level/postgraduate education (5).

** IW persons: persons employed in an IW enterprise after the agreement was enacted in 2001.

**Table 2:** **Hazard ratios** (**HR**) **of long**-**term sickness absence** (**LSA**) (>**8 weeks**) **according to potential determinants**, **women**

	**Crude**^IW**^		**Adjusted**^**a IW**^		**Crude**^**Non-IW**^		**Adjusted**^**a Non- IW**^	
	**HR**	**95%CI**	**HR**	**95%CI**	**HR**	**95%CI**	**HR**	**95%CI**
Age								
30	1	Reference	1	Reference	1	Reference	1	Reference
40	0.8	0.7-0.9	0.7	0.6-0.8	0.8	0.7-0.9	0.7	0.6-0.9
45	0.8	0.7-0.9	0.7	0.6-0.8	0.8	0.6-0.9	0.7	0.6-0.8
60	1.0	0.8-1.2	0.9	0.7-1.0	1.1	0.9-1.2	0.9	0.8-1.1
Education*								
1	2.0	1.5-2.6	2.0	1.5-2.7	2.0	1.6-2.6	2.1	1.6-2.7
2	1.7	1.4-2.1	1.7	1.4-2.1	1.7	1.4-2.2	1.8	1.4-2.3
3	1.7	1.4-2.1	1.7	1.4-2.1	1.5	1.2-1.9	1.5	1.2-1.9
4	1.5	1.3-1.8	1.5	1.2-1.8	1.4	1.1-1.7	1.3	1.1-1.7
5	1	Reference	1	Reference	1	Reference	1	Reference
Missing	1.8	1.3-2.6	1.7	1.2-2.5	2.1	1.5-3.0	2.0	1.4-2.8
Industrial classification								
Secondary industry	1.2	1.0-1.5	0.9	0.7-1.2	1.1	0.9-1.3	0.9	0.8-1.1
Tertiary industry (heavy)	0.9	0.7-1.3	0.8	0.6-1.2	0.9	0.7-1.2	0.9	0.7-1.1
Office work	1	Reference	1	Reference	1	Reference	1	Reference
Teaching sector	1.0	0.8-1.3	1.0	0.8-1.4	1.0	0.8-1.2	1.0	0.8-1.2
Health sector	1.3	1.1-1.5	1.1	0.9-1.3	1.1	1.0-1.3	1.0	0.8-1.2
Missing	1.1	0.9-1.3	1.0	0.8-1.2	1.0	0.8-1.2	0.9	0.8-1.1
Shift work, night work or rotating hours?								
No	1	Reference	1	Reference	1	Reference	1	Reference
Yes	1.5	1.3-1.7	1.3	1.1-1.5	1.5	1.2-1.7	1.2	1.0-1.5
Missing	1.3	1.1-1.5	1.1	0.8-1.5	1.3	1.1-1.5	1.4	1.0-1.9
Physical job demands								
Mainly sedentary	1	Reference	1	Reference	1	Reference	1	Reference
Involving significant walking	1.3	1.1-1.5	1.1	0.9-1.3	1.2	1.0-1.3	1.2	1.0-1.4
Heavy physical work or work involving significant walking and lifting	1.5	1.2-1.7	1.1	0.9-1.3	1.5	1.3-1.8	1.3	1.0-1.6
Missing	1.3	1.1-1.6	1.1	0.8-1.6	1.3	1.1-1.5	1.0	0.7-1.3

a Adjusted for age group, educational level, industrial classification, shift work/night work and physical job demands.

*Level of education was collapsed into five categories: Primary education/Lower secondary (1) Upper secondary education, basic (2) Upper secondary, final year/post-secondary non-tertiary education (3) First stage of tertiary education, undergraduate level (4) First stage of tertiary education, graduate level/postgraduate education (5).

** IW persons: persons employed in an IW enterprise after the agreement was enacted in 2001.

**Table 3:** **Hazard ratios** (**HR**) **of long**-**term sickness absence**(**LSA**) (>**8 weeks**) **according to potential determinants, men**

	**Crude**^IW**^		**Adjusted**^**a IW**^		**Crude**^**Non-IW**^		**Adjusted**^**aNon- IW**^	
	**HR**	**95%CI**	**HR**	**95%CI**	**HR**	**95%CI**	**HR**	**95%CI**
Age								
30	1	Reference	1	Reference	1	Reference	1	Reference
40	1.7	1.3-2.2	1.4	1.0-1.8	1.5	1.2-1.9	1.3	1.1-1.7
45	2.0	1.5-2.6	1.7	1.3-2.2	1.5	1.2-1.9	1.4	1.1-1.7
60	2.6	2.0-3.4	2.3	1.7-3.0	2.0	1.7-2.5	1.9	1.6-2.3
Education*								
1	4.6	3.3-6.5	3.3	2.2-4.9	4.4	3.2-6.0	2.9	2.0-4.0
2	3.3	2.4-4.6	2.7	1.9-3.9	3.4	2.5-4.5	2.3	1.7-3.1
3	2.6	1.9-3.5	2.4	1.7-3.3	2.8	2.1-3.7	2.3	1.7-3.1
4	1.5	1.1-2.1	1.5	1.1-2.0	1.7	1.2-2-2	1.6	1.2-2.1
5	1	Reference	1	Reference	1	Reference	1	Reference
Missing	3.7	2.2-6.1	3.4	2.0-5.8	4.7	3.2-7.0	3.8	2.5-5.7
Industrial classification								
Secondary industry	1.6	1.2-2.1	1.0	0.7-1.3	2.0	1.6-2.5	1.3	1.0-1.6
Tertiary industry (heavy)	1.9	1.4-2.7	1.2	0.9-1.8	1.8	1.4-2.3	1.1	0.9-1.5
Office work	1	Reference	1	Reference	1	Reference	1	Reference
Teaching sector	1.2	0.8-1.8	1.3	0.9-2.0	1.7	1.2-2.5	1.8	1.2-2.6
Health sector	1.6	1.2-2.1	1.3	1.0-1.7	1.5	1.1-2.0	1.2	0.9-1-7
Missing	1.3	1.1-1.8	1.0	0.7-1.3	2.0	1.6-2.4	1.5	1.2-1.8
Shift work, night work or rotating hours?								
No	1	Reference	1	Reference	1	Reference	1	Reference
Yes	1.4	1.1-1.8	1.1	0.8-1.4	1.9	1.6-2.3	1.6	1.3-2.1
Missing	1.7	1.3-2.1	2.4	1.2-4.6	1.4	1.2-1.7	1.2	0.8-1.8
Physical job demands								
Mainly sedentary	1	Reference	1	Reference	1	Reference	1	Reference
Involving significant walking	1.8	1.4-2.3	1.3	1.0-1.7	1.8	1.5-2.2	1.3	1.0-1.5
Heavy physical work or work involving significant walking and lifting	2.5	1.5-2.4	1.6	1.2-2.1	2.6	2.1-3.1	1.8	1.5-2.2
Missing	1.9	1.5-2.4	0.7	0.3—1.3	1.7	1.4-2.1	1.3	0.9-2.0

a Adjusted for age group, educational level, industrial classification, shift work/night work and physical job demands.

*Level of education was collapsed into five categories: Primary education/Lower secondary (1) Upper secondary education, basic (2) Upper secondary, final year/post-secondary non-tertiary education (3) First stage of tertiary education, undergraduate level (4) First stage of tertiary education, graduate level/postgraduate education (5).

** IW persons: persons employed in an IW enterprise after the agreement was enacted in 2001.

We made the following industry group categorisation based on the Standard Industrial Classification (SIC2002) by Statistics Norway [16]: (1) secondary industry (industry, building / construction, etc., SIC2002 codes 10 – 45), (2) tertiary industry, heavy (retail, hotel / restaurant, transport / storage / communication, SIC2002 codes 50 – 64), (3) office work (service trades / insurance, civil service, SIC2002 codes 65 – 75), (4) the teaching sector (SIC2002 code 80), (5) the health sector (health and social care, social services, SIC2002 codes 85 – 93) and (6) other/unknown (SIC2002 codes 00 – 05, 95 – 99).

The following work-related variables were included in the present analyses: *The work schedule* variable was based on the question, “*Do you do shift work*, *night work or have rotating hours of work*?” The response categories were *Yes* or *No*. *Physical job demands* were measured by the question, “*If you have paid work or do unpaid work*, *how would you describe your work*? Responses were as follows: (one cross only) *Mainly sedentary work*?; *Work involving a lot of walking*?; *Work involving a lot of walking and lifting*?; *or Heavy physical work*” The two last categories were merged into one category since only 1% of the participants had “Heavy physical work”.

### Statistical analyses

Stata/SE 11.2 was used for the analyses. Five-year risks of at least one spell of LSA in subgroups were computed with cross tabulations. Cox proportional hazards models were used to compute the LSA hazard ratios (HR) and corresponding 95% confidence intervals (CI). We adjusted for age group, educational level, industrial classification and the job exposure variables. All analyses were stratified by gender, and the main analyses were additionally stratified by IW status. We additionally performed a gender-stratified Cox regression to estimate the relative IW effect. Gender-stratified models were estimated in certain subgroups in which the results indicated heterogeneity between the IW and non-IW groups. The follow-up lasted until the end of 2005. Censoring occurred when individuals died (N=13), emigrated (N=130) or received a disability pension (N=30) or early retirement (N=603) during the follow-up period, i.e. they were no longer at risk for LSA. Of the total sample, 776 persons were censored.

## Results

A total of 3,663 employees experienced at least one LSA during the five-year follow-up period (risk 0.333). The total follow-up time was 42,044.5 person years, with an average follow-up time of 3.8 years per person. Table 1 shows the 5-year risk of having at least one spell of LSA by age, education, industrial classification and work-related factors stratified by gender and IW status. Women had a higher risk than men of experiencing an LSA. For men, there was a strong, positive relationship between age and LSA risk: For IW men aged 30, the risk was 0.138, gradually increasing to 0.312 for 60 years olds. For women, the youngest age cohort had the highest LSA risk. There was a strong education gradient for both genders, but the education gradient was more pronounced for men.

Among women in IW companies compared with non-IW companies, a larger proportion worked in the health sector (38.5% vs. 21.4%), had shift or night work (19.3% vs. 9.2%), or heavy physical work (15.8% vs. 9.1%), while a lower proportion was 60 years of age (19.6% vs. 25.5%). In addition, there were systematic differences between the IW and non-IW groups in these variables for men. For example, the proportion of IW men working in the health sector was 16.3%, whereas the proportion in non-IW men was 7.7%.

Women working in the health sector had the highest LSA risk. For men with IW employment, the highest LSA risk was observed in secondary industry, heavy tertiary industry and the health sector. For men in the non-IW group, the highest LSA risk was observed in heavy tertiary industry. Having shift work, night work or rotating hours had a strong association with the LSA risk and was strongest among men in the non- IW group (risk 0.374). The LSA risk for persons with heavy physical work and work involving a lot of walking and lifting was 30% higher in women and more than doubled in men compared with women and men with sedentary work, irrespective of IW status.

Tables 2 and 3 show the results from the Cox regression according to crude and adjusted associations for the IW and non-IW groups and women and men, respectively. The effect of age on LSA risk was stronger in men than in women in the multivariate model. The youngest women had the highest LSA risk. In the fully adjusted model, the statistically significant risk factors for LSA were low education (stronger in men, especially in the IW group), shift work/night work or rotating hours (non-significant for men in the IW group and strongest in men in the non-IW group), heavy physical work or work involving significant walking and lifting (men only and strongest in the non-IW group).

The gender-stratified Cox regressions estimating the relative IW effect showed a significant association for men in the crude analysis (HR for the IW group, with non-IW as reference: 0.88, 95% CI 0.79 – 0.99, not shown in tables). This association became statistically insignificant in the multivariate analysis (HR 0.97, 95% CI 0.86 – 1.09). In women, the HR was 1.02 (non-significant) in both the crude and adjusted analyses. We performed a separate analysis for shift working men. The adjusted HR for an LSA was significantly lower in the IW group (HR 0.67, 95% CI 0.51 – 0.88). For men in the teaching sector, IW employment appeared to have a protective effect compared with men without IW employment (Table 1). However, the corresponding adjusted HR was close to unity (0.91; 95% CI 0.56 – 1.48). We did not find any significant IW effect in other subgroups, such as men with heavy physical work or younger men. For women, the adjusted HRs were close to unity for all industries.

## Discussion

### Main findings

We have found that the most important risk factors for an LSA were shift work/night work or rotating hours (strongest in men in the non-IW group) and heavy physical work or work involving a lot of walking and lifting (men only and strongest in the non-IW group). When estimating the IW effect in each gender separately, we found no association after adjustment. However, we found an IW effect in shift working men. Our results could suggest that IW companies that employ many men in shift work have implemented relevant efforts for reducing sickness absence.

### Interpretations

Women exhibited a higher LSA risk than men, but the associations between a high physical workload and shift/night work and LSA risk were stronger for men than for women. Women working in the health sector had the highest LSA risk. The health sector is dominated by demanding work, such as hard physical work and shift and night work. Most enterprises in public industries, including the health sector, are IW enterprises. Some IW companies may have levels of high sickness absence and absenteeism because the workload is more demanding, which might have led to more interest in the IW agreement. We likely have elements of reverse causation in that high sickness absence levels in a company might have acted as an incentive to participate in the IW program. This is a plausible interpretation in cases in which the program was adopted late in the 2001–2005 period. The IW participation varies by industry. As an example, 62% of women in the health sector were in IW companies, compared with only 32% in the heavy tertiary industry (Table 1). Thus, industry may act as a confounder on the association between IW participation and LSA. IW and non-IW persons differ systematically by demographic characteristics and work environment. This is a challenge in interpreting the results. In addition, there are reasons to believe that not all enterprises with an IW agreement have achieved the IW intentions, and many non-IW businesses may have implemented sickness absence reduction efforts widely. Several measures that have arisen in the wake of the IW agreement are directed toward the entire working life, such as new sick leave rules, training of physicians and more dialogue between employers and sick-listed employees. Because no impact on the national sickness absence rates was observed by 2004, the government and social partners realised that there was a need to involve a patient’s physician because general practitioners issued approximately 80% of sickness absence certificates [1]. Since 2004, the physicians’ sickness absence certificates have been replaced by a “work ability certificate” to promote an early return to work. Sickness absence from work has gained more attention in the society as a whole after the IW agreement was implemented, which has affected the follow-up work for absentees in the entire labour market. These issues might additionally explain the lack of contrasts between the IW and non-IW groups. Many EU governments have introduced programmes aimed at encouraging long-term absentees back into work. There is still relatively limited documentation concerning the effect of workplace interventions on sickness absence [17]. The IW programme could possibly have more impact on shorter, self-certified sickness absence. However, we had no data available on this and could not explore this further.

The labour market in Norway is highly gender segregated. Studies have shown that occupational groups dominated by one gender have higher levels of absence [18], [19]. Others have found that the fact that women hold other occupations than men explain a considerable part of the differences in sickness absence [20]. Work tasks and working conditions may differ substantially between the genders or between female- and male-dominated occupations [21]. A low educational level was highly predictive of LSA, especially for men. Interventions for preventing LSA should be especially targeted to these groups.

### Strenghts and weaknesses

The strengths of this study are a relatively large study sample, combining data obtained from a survey with register-based data and a longitudinal study design. Survey-based studies of work characteristics and health have been criticised on methodological grounds for an undue reliance on self-reported outcome measures and not addressing information bias [22]. The basic source of a dependent error [23] in such studies is usually a normal variation in certain personality traits, but the error may additionally be in more transitional moods in the study population or inadequate measurement tools. Registry-based rates of physician-certified sickness absence may be a more accurate measure of health status than self-reports, which are often biased [24]. The major precaution that should be taken to eliminate bias from dependent error is to break the bond between information on exposure and outcome by gathering data from two separate sources [25], which is what was done in this study. Additionally, the data on educational level were objective information from public registries. The low participation rate (46%) in HUBRO may have led to the self-selection of healthy subjects into the study. The predictors of participation and magnitude and direction of the non-response bias in prevalence estimates and association measures have been investigated based on information from all 40,888 invitees to the Oslo Health Study. The potential selection bias was studied by linking register-based data from Statistics Norway on demographics, lifestyle and social security benefits to the entire study population. Unhealthy persons participated to a lesser degree than healthy individuals, but the social inequality in health by different socio-demographic variables appeared unbiased [26]. In addition, the response rate to the supplementary questionnaires on working conditions was lower in subgroups with worse health [27]. Because poor health often is associated with a poor work environment, this finding may imply a lower response among subjects with poor work environments and thus conservative estimates of the effects of working conditions on LSA.

## Conclusions

Despite the great transitions working life has undergone in recent decades, with an increase in automation and a change to more sedentary work, shift, physical and strenuous work are still important predictors of sickness absence. Due to the often-observed gender differences in LSA, future research should include both genders to explore these matters further. Future research should additionally focus on the work environments that might be responsible for these factors.

## Abbreviations

CI: Confidence Interval; FD-Trygd: The Historical Event Database of Statistics Norway; HR: Hazard Ratios; HUBRO: The Oslo Health Study; IW: Inclusive working life; LSA: Long-term (>8 weeks) continuous sickness absence; SEP: Socio-economic position.

## Competing interests

The authors declare that they have no competing interests.

## Authors’ contributions

LF had the original idea for the study and its design. LF and HMG carried out analyses. HMG, PK, BC, ISM and KS contributed to the statistical strategy and contributed to the interpretation and discussion of findings. LF drafted and completed the manuscript. All authors read and approved the final manuscript.
